# Beyond the division of multiple sclerosis into different subgroups: The Concept of Connectomopathy

**DOI:** 10.22088/cjim.15.3.370

**Published:** 2024-08-01

**Authors:** Abdorreza Naser Moghadasi, Nasim Rezaeimanesh

**Affiliations:** 1 Multiple Sclerosis Research Center; Neuroscience Institute, Tehran University of Medical Sciences, Tehran, Iran

**Keywords:** Multiple Sclerosis, Connectomopathy, MS phenotypes

## Abstract

Multiple Sclerosis (MS) pathophysiologically is a dynamic and progressive disease that involves all parts of central nervous system. This widespread involvement of the CNS has paved the way for proposing a new theory in MS in which MS is considered as a connectomopathy. Connectomopathy is a new concept describing the diseases in which not only the brain connectome is completely and extensively damaged, but the defective connectome itself can also become a breeding ground for the disease’s progression. Connectomopathy provides a dynamic picture of MS. Since each person’s connectome is unique to him/herself, so MS patients’ connectomopathy varies from one to another. This variety not only challenges the classification of MS into different phenotypes, but also emphasizes the need for providing a personalized approach for the treatment of these patients.

The variety of clinical signs of multiple sclerosis (MS) with different patterns at the onset and during the progression of the disease has divided MS into different subgroups. Accordingly, of these, the most famous classification, which is also the oldest one, categorizes MS disease into the following four subgroups: relapsing-remitting MS (RRMS), secondary progressive MS (SPMS), primary progressive MS (PPMS), and progressive relapsing MS (PRMS). The difference among these subgroups is in the roles of attacks, their recurrence and the progression of the disease (1). However, the difference among these phenotypes in terms of the pathophysiological basis of this disease is not very clear yet. Correspondingly, these cases led to the introduction of a new classification of MS in 2014. 

Lublin et al. in their research based their previous works on the same two main concepts of relapses and disease’s progression, and instead of using the old terms, based on the disease’s activity and progression classified the progressive type of MS into the following four subgroups: active with progression, active without progression, inactive and with progression, and inactive and without progression. Relapsing MS was also divided into the active and inactive subgroups (2). In all these divisions, the important point about MS that has always been of interest to researchers studying in this field was the amount of attacks and progression in this disease. Tremlett et al. in their study found that seizures could affect the disease’s progression in short-term, but no such effect was observed in long-term (3).On the other hand, Paz Soldán et al. found that the rates of attacks before and after the onset of the clinical phase of the disease’s progression could be directly related to the time and the severe disability (4). In another study by Rudick et al., some factors involved in patients' disability were assessed in an 8-year period of following up those patients who participated in a clinical trial called the Multiple Sclerosis Collaborative Research Group (MSCRG) study.

Moreover, this clinical trial evaluated the recurrence and disability rates in patients receiving intramuscular interferon compared with the placebo group. Thereafter, they re-analyzed the results obtained from the follow-up of these patients for 8 years and found that the rate of attacks is a determining factor in the degree of disability in the long run (5). Therefore, attacks and progression cannot be separated, and any model proposed to describe MS must clearly demonstrate this relationship.

**Convergent models used to explain different MS phenotypes**

 The MS topographic model proposed by Krieger in 2016 pursued the same goal. In this study, by presenting this model, he attempted not only to establish a dynamic connection between the attacks and the progression of the disease, but also to unify practically all forms of MS in terms of this model. Moreover, his model, which was proposed based on the five main items, including injury caused by disease attacks, severity of attacks, its recovery rate, number of attacks, and disease progression rate (6), had two general concepts of symptomatic or asymptomatic plaques and disease progression. Correspondingly, this explains all the seemingly distinct types of MS, and shows how all these distinct subgroups, including RRMS, SPMS, PPMS, clinically isolated syndrome (CIS), and radiologically isolated syndrome (RIS) can be explained using the topographic model of MS.

According to this model, the central nervous system (CNS) is such a pool full of water, which can be divided into three main parts with different depths. In this regard, the first one includes the spinal cord and optic nerve, which have less depth compared to the other areas of the CNS. In addition, the second part consists of the brainstem and cerebellum, which are moderately deeper than the other areas, and the final part as the hemispheres, which are the deepest part of the pool. As well, plaques from MS attacks are the topographic peaks from the bottom of the pool. The amount of water in the pool indicates its neurologic functional capacity. Neurologic functional capacity is the brain reserve involved in the amount of compensatory mechanisms, and to some extent, it can prevent the marking of the damaged areas of the brain.

It was shown that each person's brain reserve also changes under the effect of different conditions such as illness, environmental stimuli, education, and aging. Additionally, it should be noted that various factors such as brain size and personality traits are involved in both the amount and content of brain reserves (6-8). Notably, in this model, the progression of the disease shows itself as a gradual decrease in pool water. 

According to this model, the entire central nervous system is damaged during MS, which in particular manifests itself in more reduction of brain’s reserve and atrophy. In addition, this fact that the whole brain is damaged during MS can be inferred from numerous imaging findings (9). It should be noted that by the use of traditional imaging techniques, this general conflict cannot be well-illustrated. However, new imaging techniques are able to show these general and widespread involvement in the brains of people with MS. In a previous study, De Santis et al. used a new method called a multiple-b-value, high angular resolution multi-shell diffusion MRI protocol with various diffusion times, to examine the brains of patients with MS. Using this method, they found some microstructural changes in the whole brain of these patients (10). Moreover, Lommers et al. in their study examined the normal appearing brain tissue using Quantitative MRI (qMRI) and a multiparameter mapping (MPM) protocol and showed a diffuse reduction in myelin and / or iron content (11). Other studies have also shown that this widespread change begins at the onset of the disease (12). In addition, this extensive change can progressively be observed during the course of the disease. By performing one year cohort studies on how to change MRI parameters using the advanced methods, it was shown that despite no clinical change in patients, axonal pathology widely persists in normal-appearing white matter (13). Correspondingly, this represents a dynamic and progressive change in the overall structure of the brain.

**Connectomopathy**

This widespread involvement of the CNS has paved the way for proposing a new theory in MS in which MS is considered as a connectomopathy. Accordingly, this refers to those diseases in which not only the entire connectome is involved, but also the affected connectome acts as a bedrock for both the growth and progression of the disease (14). In this regard, the connectome refers to the set of connections and neural pathways in the CNS (14). As mentioned earlier, imaging studies have previously indicated extensive damage to the CNS of patients with MS. However, this damage does not end in a permanent, non-progressive disorder. In this disease, brain connectome does not work correctly and can cause the progressive involvement of connections and different pathways of CNS. In this regard, connectomopathy is a hidden aspect of the disease. 

If a disease like MS causes damage to the tissues of the CNS due to leading to some problems with the immune system, the entire brain connectome would be damaged as a result of the way through which it is destroyed. Nevertheless, this damage is not static and fixed, so after removing the immunological damage, the process of the destruction of the CNS would not stop. Rather, the whole connectome can act as an autonomous and self-sufficient system and then help in advancing the process of destruction. Thus, the progression of the disease is considered as one of the most important aspects of MS, and as observed, despite the fact that the disease is clinically stable, we mostly face the progression of pathology in images obtained from the imaging devices. The same dynamic and generalized trend of the disease makes it impossible to differentiate the types of phenotypes based on their clinical characteristics. Connectomopathy is a dynamic process for MS, but due to progressive changes, it does not have any static state. 

Moreover, we can discuss about phenotypes with specific characteristics in MS when the disease does not have such dynamic and changing appearances. Undoubtedly, deeper analysis of imaging data can help us in gaining a better understanding on MS.

**Models of radiological data analysis of MS**

From the perspective of connectomopathy, there is a direct and two-way relationship between the onset and progression of the disease in the topographic model of the disease. Therefore, to better understand the nature of MS and then to explain the different clinical phenotypes, we need to pay more attention to the concepts of connectome and multiple brain connections. For this purpose, we need new data as well as different in-depth analyses of imaging data. Therefore, we must not only show the brain connectome, but also show the benefit of the use of analytical methods to present the features of autonomy and the dynamics of this connectome in MS. Although to better analyze imaging data, increasing efforts have been made, no such thing that can accurately demonstrate connectomopathy has been presented so far. Recently, a model has been proposed in a study by Eshaghi et al. based on the imaging findings to classify MS (15).

 In this model, instead of relying on clinical concepts with no clear border with each other, by analyzing MRI data obtained from 6322 patients with MS, three types of radiological phenotypes have been identified. Thereafter, these phenotypes were divided into three main groups depending on how the lesions progress radiologically as follows: cortex-led, normal-appearing white matter-led, and lesion-led. Interestingly, a direct relationship was found among these phenotypes, clinical symptoms, and response to treatment. In this regard, although this model provides us with more details on MS phenotypes based on imaging data, it is a static model and cannot show the fluidity hidden in the concept of connectomopathy.

**The need to provide a model based on the concept of connectomopathy**

While all these efforts were made to help us in better understanding MS, there is still a long way to show MS as a connectomopathy. As mentioned earlier, connectomopathy presents a fluid, autonomic image of the cerebral connections system in MS, as well as an autonomous system to accelerate the neurodegenerative process. In fact, the algorithm that should be used to analyze the imaging data is a dynamic one that not only analyzes the available data, but it can also use static imaging data offered by conventional MRIs for its dynamic context. In addition, these algorithms should be able to both create and build new algorithms based on how connectomopathy progresses.

Such an algorithm can predict the progress and self-destruction of the connectome based on the initial MRI data and then show them. Only after that, a true view of the CNS in MS can be provided.

**Classification of MS phenotypes based on the new model**

If such an algorithm was researched, what would be considered as the classification of central nervous system involvement phenotypes in MS? Accordingly, it can be said that each phenotype, both clinically and radiologically, shows a specific trend of how the CNS is affected by the disease. However, if we look at the disease with the concept of connectomopathy, the border between different phenotypes would be blurred, indicating that each one of them is in fact presented as a damage of the connectome with different degrees. From this point of view, each MS patient can be considered as a unique case. While MS is defined as a connectomopathy triggered by a dysfunction of the immune system, anything that can influence a person’s connectome will be consequently involved in the MS phenotype. Of note, brain connectome is resulted from a two-way communication between the brain and the surrounding world, which is made up of a person’s genetics, the environment in which he/she lives, the form of his/her nutrition, his/her education, and his/her activities. All of these factors will affect the neural pathways in the brain, and consequently, the shape of the person’s connectome.

In other words, depending on a person’s experiences and genetic background, the shape of his or her connectome would vary. The same thing will make her/his MS disease’s state unique. From a therapeutic point of view, the same issue makes the personalized medicine an inevitable issue (16). Although science still is not able to achieve this in the field of MS treatment, it is necessary to pay more attention to such a category to provide more effective treatments.

## Conclusion

MS pathophysiologically is a dynamic and progressive disease that manifests itself as a connectomopathy. In this disease, not only the brain connectome is widely damaged, but this damaged connectome itself can also become a bedrock for the progression of the disease afterward. Therefore,to better understand the mechanism of the disease, it is necessary to design some algorithms that can change themselves dynamically to show the progression of the disease in the entire central nervous system.
